# Design, Modeling, and Performance Analysis of a 2D Non-Resonant Flexible Vibration-Assisted Polishing Device with a Series Large-Displacement Parallel Mechanism

**DOI:** 10.3390/mi17101194

**Published:** 2026-10-15

**Authors:** Xiaoming Zhang, Yan Gu, Bin Fu, Xuesong Wang, Zisu Xu, Yuan Xi, Weidong Zhou, Silin Liu

**Affiliations:** 1 Jilin Provincial Key Laboratory of Micro-Nano and Ultra-Precision Manufacturing, School of Mechatronic Engineering, Changchun University of Technology, Yan’an Ave 2055, Changchun 130012, China; zxm20011019@163.com (X.Z.); 17748242912@163.com (X.W.); 15568607008@163.com (Z.X.); 13944475509@163.com (Y.X.); zwd13756458187@163.com (W.Z.); 13756896298@163.com (S.L.); 2 Jilin Provincial Key Laboratory of International Science and Technology Cooperation for High Performance Manufacturing and Testing, School of Mechatronic Engineering, Changchun University of Technology, Yan’an Ave 2055, Changchun 130012, China

**Keywords:** compliant mechanism, vibration-assisted polishing, finite element analysis, compliance matrix method, flexure design

## Abstract

To address the issues of graphite flaking and interfacial microcrack propagation during conventional polishing of multiphase heterogeneous materials such as ductile cast iron, this study designed and validated a two-dimensional non-resonant flexible vibration-assisted polishing device (RVAPD) based on a series–parallel structure combined with a Scott Russell mechanism. The device decouples spatial motion from mechanical transmission through an irregular flexible hinge network. A theoretical model is established using the flexible matrix method and Lagrange equations, and static and dynamic simulations are conducted using ABAQUS finite element software. The simulation results show that the input and output stiffness values are 19,524 N/mm and 8197 N/mm, respectively. Experimental measurements revealed a natural frequency of 1120 Hz, a maximum output displacement of 12.58 μm, an output stiffness of 7200 N/mm, a static response delay of only 114 ms, and a positioning error below 2.76 μm, confirming the device’s high stiffness, wide bandwidth, and precise motion control capabilities. Polishing experiments indicate that 200 Hz provided the best surface-quality performance among the tested excitation frequencies, at which the surface roughness Sa decreased from 0.049 μm without vibration to 0.036 μm, while the maximum height Sz decreased from 1.927 μm to 0.983 μm. The polishing performance slightly deteriorated at 300 Hz, with Sa increasing to 0.043 μm. These results indicate that the tested excitation conditions can influence the surface-quality characteristics of the polished ductile cast iron. This research provides a basis for non-resonant vibration-assisted polishing of multiphase heterogeneous alloys.

## 1. Introduction

Ductile cast iron (DCI) has been widely used for structural components, such as crankshafts and wind turbine main shafts, subjected to heavy and alternating stresses [1,2]. This is due to its internal spherical graphite, which can effectively mitigate stress concentrations. DCI also possesses high tensile strength and excellent fracture toughness. However, as a typical multiphase heterogeneous material, there is a significant mismatch between the physical and mechanical properties of the graphite phase and those of the metal matrix within DCI. During conventional mechanical processing, the intense thermo-mechanical coupling effect not only easily induces residual tensile stress on the surface but also causes the spherical graphite nodules to detach under compressive and shear loading (nodule pull-out), accompanied by micro-fractures in the matrix and plastic smearing [3,4,5]. These surface and subsurface microscopic defects destroy the continuity of the material structure, making these defects potential initiation sites for fatigue cracks during service [6,7]; at the same time, the rough micro-pits will destabilize the Hydrodynamic Lubrication Film, exponentially accelerating the contact fatigue and friction wear process of the components [8,9]. To prevent the evolution of microscopic defects into macroscopic structural failure, precise surface integrity control is indispensable. Vibration-assisted polishing technology, by introducing high-frequency dynamic vibration, can effectively suppress graphite peeling and matrix damage, improve surface morphology, and enhance material performance.

Against this background, vibration-assisted machining technology (VAM) has become an effective intervention method for suppressing surface micro-defects and improving processing efficiency by actively changing the contact mode of the tool or abrasive particles. According to the relationship between the operating frequency and the natural frequency of the device, VAM can be strictly classified into resonant and non-resonant types. The resonant type is based on the self-excited vibration principle (mostly concentrated in the ultrasonic frequency range), with ultrasonic vibration-assisted cutting (UAC) as a typical representative. Since the pioneering research by Kikukawa et al., a large body of literature has confirmed that this technology uses high-frequency, low-amplitude vibrations to periodically separate the tool from the workpiece, significantly shortening the friction time, reducing the cutting force, and optimizing the surface integrity. However, the application range of modern VAM technology has expanded to a broader frequency range, covering resonant and non-resonant composite processing methods in the high-frequency and low-to-medium-frequency bands [10]. Different from resonant processing at a fixed frequency, the non-resonant-type VAM relies on the forced vibration mechanism, and its effective working frequency band is usually limited to the medium- and low-frequency range (≤11 kHz). This type of method has been widely applied in various mechanical processing techniques such as grinding, turning, and milling. Previous studies have fully confirmed that introducing non-resonant forced vibration can effectively extend the tool life [11], precisely synthesize complex microstructures [12], and dynamically control surface texture features [13]. In the polishing field, vibration-assisted polishing (VAP) reconstructs the kinematic trajectories of abrasive particles and serves as an important approach for improving the surface integrity of hard, brittle, and multiphase composite materials. In a specific mode, resonant ultrasonic vibration-assisted polishing (UVAP) has been demonstrated to effectively improve processing quality. Suzuki et al. [14] successfully processed optical molds with high numerical aperture using two-axis ultrasonic vibration; Zhao et al. [15] experimentally confirmed that ultrasonic waves can significantly reduce the friction force on the SiC surface. Although UVAP has established an extremely high technical benchmark, given the thermal effects and ultrasonic cavitation associated with high-frequency operation [16], as well as the closed-loop control requirements for complex spatial cutting trajectories, developing non-resonant, medium-to-low-frequency vibration-assisted equipment with continuously adjustable operating frequencies has become an inevitable direction for the development of the field of precision manufacturing. Non-resonant vibration-assisted machining not only expands the degrees of freedom of macroscopic motion trajectories but also provides mechanical boundaries for rheological control under multi-physical field interactions. Chee et al. [17] experimentally demonstrated the effectiveness of two-dimensional low-frequency vibration polishing. Li et al. [18] first established a material-removal function model for two-dimensional non-resonant polishing. Furthermore, the multi-field coupling effects induced by non-resonant vibrations have opened new pathways for processing multiphase materials: Zhao et al. [19] further revealed the dynamic amplification effect of periodic viscous waves on fluid dynamic pressure. Achieving these complex hydrodynamic and tribological effects requires precise motion control. To this end, extensive structural improvements have been made to existing physical mechanisms. For instance, Yun et al. [20] developed a walking-type piezoelectric actuator that achieved alternating elliptical motion of driving feet through out-of-phase driving of the dual stators, enabling wide-speed-range regulation from 0.42 to 24.6 mm/s across frequencies of 1–15,000 Hz, with forward and reverse positioning resolutions of 144 and 152 nm, respectively, completely eliminating backward drift. Le et al. [21] emulated the biomechanical characteristics of spider legs to develop an XY dual parallel mechanism, leveraging the stiffness and flexibility of leaf-spring balancing systems to achieve high-fidelity spiral trajectory output, with a maximum workspace of 2.62 mm × 2.62 mm. Subsequently, based on the joint “energy storage” mechanism of grasshopper hind legs [22], the team proposed a 2DOF bio-inspired compliant mechanism with self-amplifying features, achieving an amplitude gain of 4.53 without relying on conventional displacement amplifiers, with a maximum operating frequency up to 548 Hz. Meng et al. [23] introduced an exponential hybrid longitudinal asymmetric flexible hinge (EH-LAFH), which employs a longitudinally asymmetric design to create unequal stiffness on both sides of the hinge, enabling a “slow advance, fast retreat” displacement response when driven by sinusoidal excitation in piezoelectric stick–slip actuators. This design provided novel insights into the application of flexible hinges in variable-stiffness mechanisms.

Although our previous work developed a rotary vibration-assisted polishing device (RVAPD) based on the Hoecken linkage mechanism and demonstrated its feasibility for polishing SiC ceramics [24], the previous transmission topology was primarily designed for rotary vibration generation in hard and brittle materials. In the previous design, the PZT-driven motion was transmitted through a linkage-based Hoecken mechanism to generate rotation of the central platform. The experimentally measured output stiffness was 4454 N/mm, while the theoretical and finite element output stiffnesses were 6145.8 N/mm and 6353.2 N/mm, respectively [24]. In contrast, the present study introduces a series–parallel compliant transmission architecture combined with a Scott Russell mechanism. The proposed topology replaces the original linkage-dominated transmission path with a compliant transmission network and redistributes the structural stiffness between the input and output sides. The theoretical output stiffness of the proposed device is 8856 N/mm, and the finite element value is 8197 N/mm, representing increases of 44.1% and 29.0%, respectively, relative to the corresponding values of Ref. [24]. More importantly, the experimentally measured output stiffness reaches 7200 N/mm, which is approximately 61.6% higher than the 4454 N/mm obtained in our previous design. This improvement is achieved without simply increasing the overall input stiffness: the theoretical input stiffness decreases from 51,052 N/mm in Ref. [24] to 23,800 N/mm in the present design, while the output stiffness increases from 6145.8 N/mm to 8856 N/mm. This indicates a deliberate redistribution of compliance, providing sufficient input compliance for PZT actuation while maintaining higher stiffness at the polishing end. The proposed device therefore differs from Ref. [24] not only in the use of a Scott Russell mechanism, but more fundamentally in its series–parallel compliant transmission topology and stiffness allocation strategy. In addition, whereas Ref. [24] experimentally demonstrated rotary vibration-assisted polishing of SiC ceramics, the present study focuses on multiphase ductile cast iron, for which higher resistance to polishing-induced external disturbances is required. A detailed comparison is shown in Table 1 These structural and application-level differences constitute the primary novelty of the present work.

## 2. Design and Working Principle

**Figure 1:**
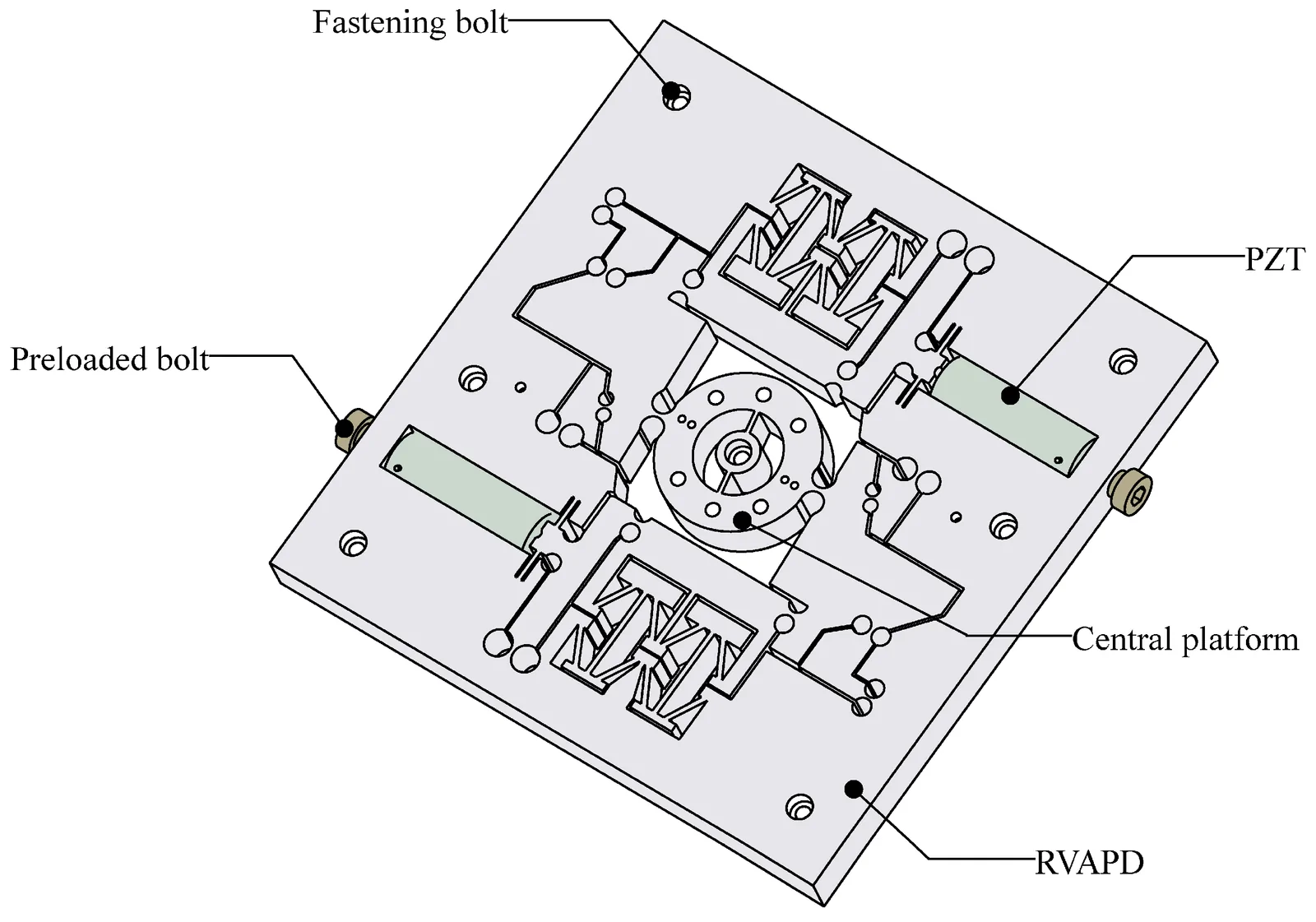
The structural composition of the RVAPD.

This polishing device mainly consists of the RVAPD core body, connecting plates, PZT actuators, and pre-tightening bolts. As shown in Figure 1, the pre-tightening bolts apply an initial preload to the PZT actuator, ensuring that the equipment can output precise movements. Two groups of PZT actuators are arranged in parallel to provide the required displacement and force for rotating the central platform. The overall layout of this device is compact, effectively reducing the weight while enhancing the natural frequency of the system. Moreover, the thickened hinge design not only enhances the overall rigidity of the body and improves its dynamic characteristics but also effectively reduces the maximum stress experienced by the equipment during long-term operation.

**Figure 2:**
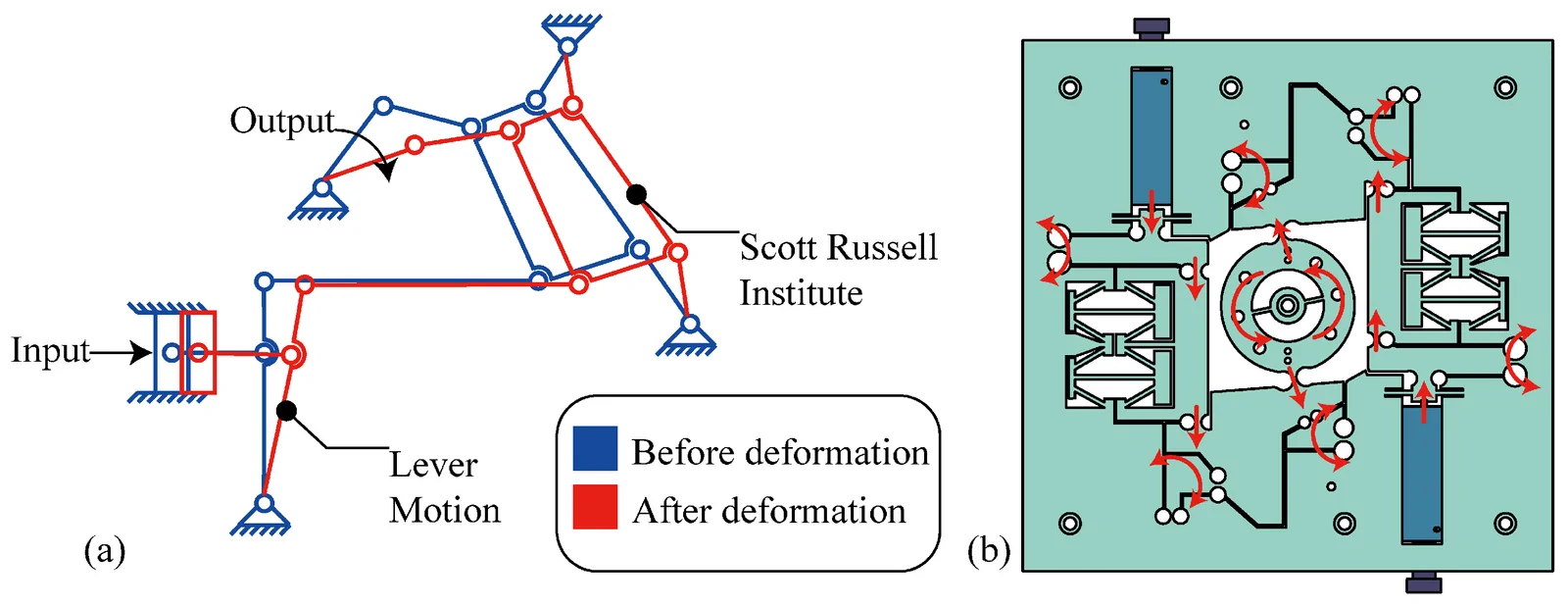
The working principle of the RVAPD: (**a**) Schematic diagram of the mechanism movement. (**b**) Schematic diagram of displacement transmission.

The transmission mechanism adopts the Scott Russell Straight-line Mechanism (SRM) and connects two identical parallel mechanisms in series to reduce transmission losses and axial drift of the hinge at the connection end. The input stiffness of this guiding mechanism should be moderate, the output stiffness should be high, and the overall size should be small, as shown in Figure 2a. The displacement amplification module mainly adopts a single-stage lever design. Since the dimensions and geometry of the lever directly affect the displacement transmission performance [25], this design specifically utilizes this single-stage lever structure to achieve a greater transmission displacement.

When the PZT generates input displacement, it pushes the lever and counterweight block. Through the series of large-stroke parallel components and the Scott Russell linear structure, the linear displacement generated by the piezoelectric actuator is converted into the rotational motion of the central platform. Additionally, thanks to the overall symmetrical layout and the coordination of the intermediate connecting rod, the rotation of the central component about the *Z*-axis can be achieved through two transmission paths. The specific stroke transmission route of this device is shown in Figure 2b. Although a part of the motion distance is lost during the transmission process, according to the design concept of the equipment, the rigidity and flexibility of the structure are inversely proportional, and the enhanced rigidity is precisely the key to maintaining the accuracy of the transmission [26]. Considering the actual processing needs, this design ensures that the inherent frequency of the equipment exceeds 1000 Hz, and comprehensively evaluates the magnitude and distribution position of the ultimate force, so as to optimize the output stroke and structural stiffness.

The input motion of the proposed RVAPD is the linear displacement generated by the two symmetrically arranged PZT actuators. The displacement is first transmitted through the lever and the series large-stroke compliant parallel mechanism and is subsequently converted by the Scott Russell mechanism into the rotational motion of the central platform about the *Z*-axis. Owing to the symmetric transmission paths on both sides of the platform, the two input displacements jointly drive the central platform and generate the required in-plane vibration trajectory at the polishing interface. In this study, the term “two-dimensional motion” refers to the planar motion trajectory at the polishing interface rather than two independent rotational degrees of freedom of the central platform. The tangential displacement at the platform edge is therefore used as the measurable output displacement to characterize the vibration motion.

## 3. Static and Dynamic Model Analysis

To clarify the relationship between the mechanical structure of the RVAPD and its force performance and to determine the key dimensions and performance limits of the device, theoretical analyses of its static and dynamic characteristics are required. At the static compliance analysis level, given the minute deformation characteristics of the flexure hinges within the RVAPD and its complex spatial topology, this study does not adopt the pseudo-rigid-body model method [27], which is commonly used for large-deflection analysis, but instead adopts the flexibility matrix method [28]. This method is based on the concept of finite element discretization. This approach parameterizes the flexure hinges as equivalent compliance elements while treating the remaining components as rigid bodies, thereby enabling analytical solutions for the deformation of the multi-degree-of-freedom system of the complex series–parallel structure. At the dynamic modeling level, this section establishes the dynamic model using the Lagrange equation. By establishing the relationship between kinetic and potential energies through generalized coordinates, and extracting the natural frequency under a specific boundary, the effects of non-conservative forces are considered, and the dynamic control equation of the system is rigorously derived. Ultimately, the above modeling process provides theoretical solutions of the input/output stiffness matrix and natural frequency of the RVAPD, establishing a mathematical benchmark for the subsequent stiffness matching of the equipment structure and the safe selection of non-resonant working frequency bands.

### 3.1. Static Model Analysis

#### 3.1.1. Flexibility Matrix Method

To meet the dual requirements of the RVAPD during processing, namely large stroke displacement and sub-micron-level guiding accuracy, this device adopts a transmission architecture combining a heterogeneous flexure hinge network. Specifically, leaf-spring flexure hinges (LSFHs) are employed to exploit their distributed flexibility characteristics to meet the requirements of large-scale translational displacement; at the same time, right circular flexure hinges (RCFHs) are connected in series, relying on their combined flexibility characteristics to maintain the precise rotation center and provide extremely high stiffness against parasitic motion, thereby suppressing parasitic displacement. In the static mechanical modeling, the elastic deformation of the flexible elements is mapped based on Screw Theory. The spatial micro-deformation at the hinge end is defined as a Kinematic Screw $U={\left[u_{x},u_{y},u_{z},\theta_{x},\theta_{y},\theta_{z}\right]}^{T}$, and the generalized force system acting on it is defined as a Load Screw $W={\left[F_{x},F_{y},F_{z},M_{x},M_{y},M_{z}\right]}^{T}$. By introducing the Characteristic Compliance Matrix C, the control equation U=C·W is established, thereby obtaining an analytical linear relationship between the applied load and the spatial multi-degree-of-freedom deformation, laying a mathematical foundation for the subsequent overall static flexibility calculation of the system.

**Figure 3:**
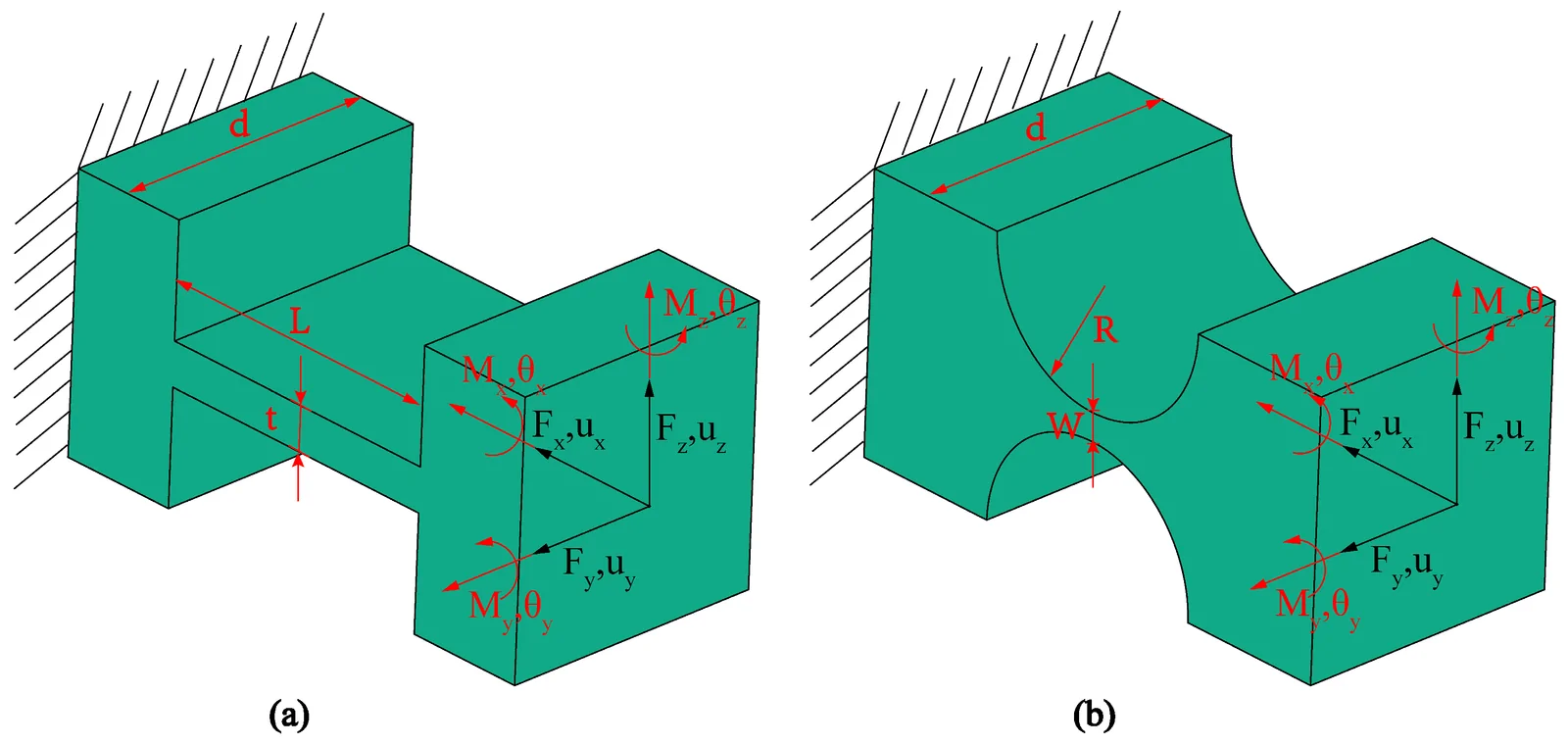
Key dimensions of the hinge and local coordinate system. (**a**) Local coordinate system of RCFHS. (**b**) Local coordinate system of LSFHS.

Figure 3 indicates the specific dimensions of the selected hinges and their local coordinate systems. The relationship between the deformation at the end of the flexible hinge and the applied external force can be calculated using Formula (1), where the parameter C in the formula is entirely determined by the geometric dimensions of the flexible hinge itself:

(1) $$\left[\begin{array}{c} u_{x}\\ u_{y}\\ \begin{array}{c} u_{z}\\ \theta_{x}\\ \begin{array}{c} \theta_{y}\\ \theta_{z} \end{array} \end{array} \end{array}\right]=\left[\begin{array}{cccccc} C_{11} & 0 & 0 & 0 & 0 & 0\\ 0 & C_{12} & 0 & 0 & 0 & C_{15}\\ 0 & 0 & C_{33} & 0 & C_{24} & 0\\ 0 & 0 & 0 & C_{44} & 0 & 0\\ 0 & 0 & C_{42} & 0 & C_{55} & 0\\ 0 & C_{51} & 0 & 0 & 0 & C_{66} \end{array}\right]\left[\begin{array}{c} F_{x}\\ F_{y}\\ F_{z}\\ M_{x}\\ M_{y}\\ M_{z} \end{array}\right]$$

**Figure 4:**
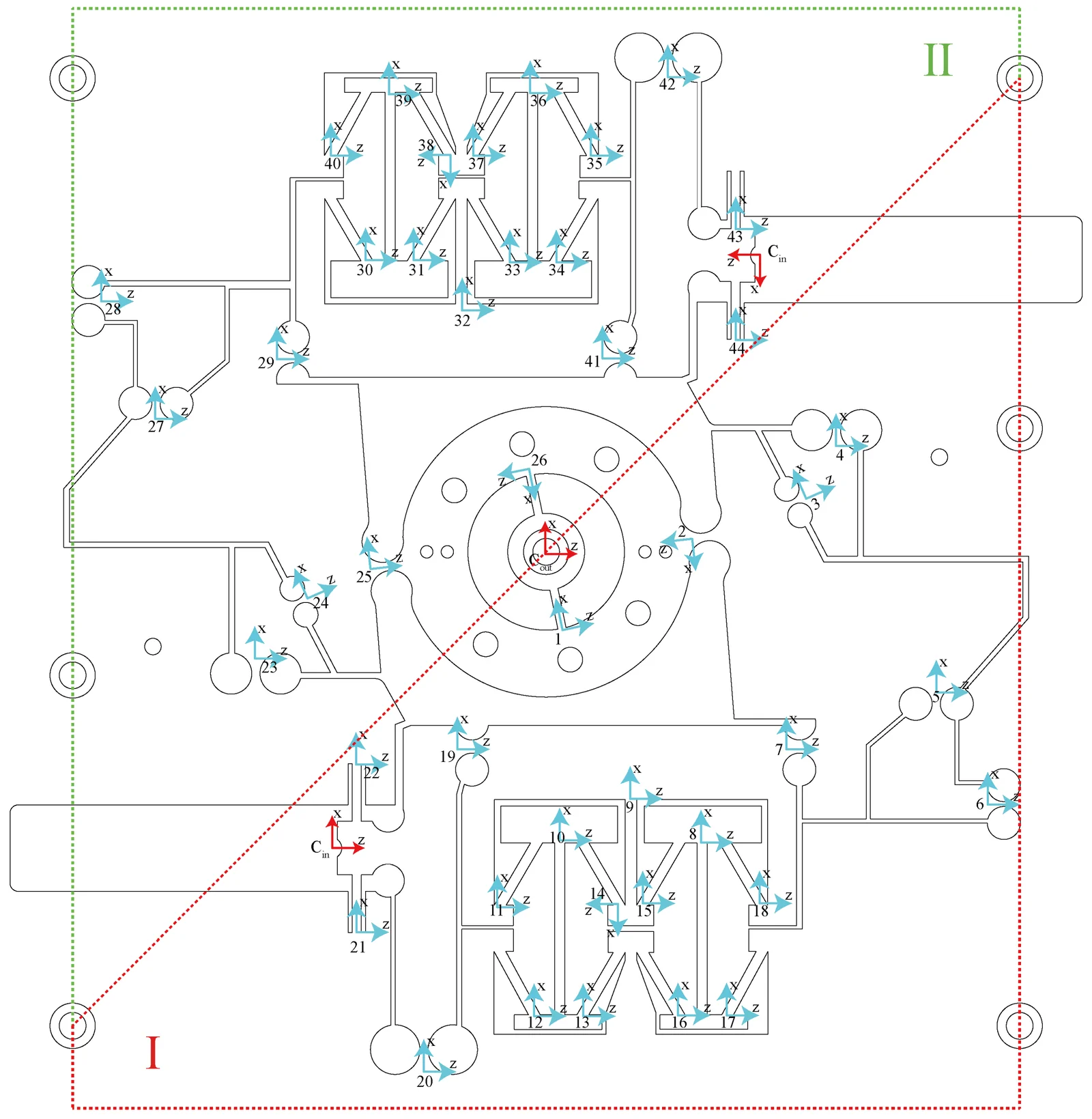
Local and global coordinate systems (Red and blue coordinate systems denote the global and local coordinate systems, respectively; I and II denote the two half-regions obtained by symmetry simplification of the device.).

Given that the RVAPD configuration possesses strict central symmetry, in the theoretical modeling stage, the substructure dimensionality reduction and decoupling strategy can be adopted. As shown in Figure 4, the entire device can be physically discretized along the symmetry center into structurally equivalent substructures, I and II. Based on this symmetry fact, the static analysis can be reduced to a single substructure: once the parallel and series mapping network of each flexure hinge within the unilateral substructure is established through the flexibility matrix method, the global properties can be deduced using the symmetry transformation operator. In terms of coordinate system definition, the input local coordinate system is strictly fixed to the driving action node of substructure I, and the output local coordinate system is fixed to the end effector center of substructure II. Subsequently, based on the displacement coordination and force balance boundary conditions at the connection nodes of the two substructures, their local stiffness matrices are equivalently reconstructed in series. This decoupling mapping process not only accurately derives the global input and output stiffness of the entire system, but also significantly reduces the computational redundancy of the high-dimensional static equations.

#### 3.1.2. Stiffness Modeling

In the static and dynamic characterization of the RVAPD, the stiffness distribution of the compliant transmission system directly affects both the force transmission capability and the displacement response of the PZT-driven mechanism. It also determines the equivalent stiffness used in the subsequent dynamic model and, consequently, the undamped natural frequency of the device [29]. Therefore, the input and output stiffnesses of the proposed mechanism were derived based on the flexibility matrix method described in Section 3.1.1. Owing to the central symmetry of the RVAPD, the complete mechanism can be divided into two structurally equivalent substructures, denoted as substructures I and II, as shown in Figure 4. The local coordinate system of each flexure hinge was first transformed into the corresponding global coordinate system according to its spatial orientation. After this coordinate transformation, the compliance matrices of all flexure hinges involved in the same transmission path can be combined according to their actual series and parallel relationships. This treatment ensures that the compliance matrices participating in the subsequent matrix operations are expressed in a consistent coordinate system.

At the input side, substructures I and II are symmetrically arranged and subjected to the corresponding input loads simultaneously. Therefore, from the perspective of the input node, the two substructures form a parallel compliance configuration. Let $C_{I}^{O_{in}}$ and $C_{II}^{O_{in}}$ denote the equivalent input compliance matrices of substructures I and II, respectively. According to the stiffness superposition principle for parallel mechanisms, their equivalent global input compliance matrix $C_{in}$ can therefore be expressed as

(2) $$C_{in}={\left[{\left(C_{I}^{O_{in}}\right)}^{-1}+{\left(C_{II}^{O_{in}}\right)}^{-1}\right]}^{-1}$$

The equivalent compliance matrices $C_{I}^{O_{in}}$ and $C_{II}^{O_{in}}$ are further obtained by following the actual series–parallel topology of the flexure hinges within each substructure. For a series connection, the compliance matrices are directly added because the same load is transmitted through the connected elements and the total deformation is the sum of the individual deformations. For a parallel connection, the corresponding stiffness matrices are first superimposed and then transformed back into the equivalent compliance matrix. Based on these relationships, the equivalent input compliance matrices of substructures I and II are given by

(3) $$C_{I}^{O_{in}}={\left\{\begin{array}{c} {\left[{\left(C_{22}^{-1}+C_{21}^{-1}+C_{20}^{-1}\right)}^{-1}+C_{19}+C_{7}\right]}^{-1}\\ +{\left(C_{3}+C_{4}\right)}^{-1}+{\left(C_{6}+C_{5}\right)}^{-1} \end{array}\right\}}^{-1}+C_{2}$$

(4) $$C_{II}^{O_{in}}={\left\{\begin{array}{c} {\left[{\left(C_{43}^{-1}+C_{44}^{-1}+C_{42}^{-1}\right)}^{-1}+C_{41}+C_{29}\right]}^{-1}\\ +{\left(C_{28}+C_{27}\right)}^{-1}+{\left(C_{23}+C_{24}\right)}^{-1} \end{array}\right\}}^{-1}+C_{25}$$

Once the equivalent global input stiffness matrix $C_{in}$ is obtained from Equation (2), the corresponding global input stiffness matrix can be calculated based on the inverse relationship between strain and stiffness, as shown in Equation (5):

(5) $$K_{in}={\left(C_{in}\right)}^{-1}$$

The stiffness component corresponding to the principal driving direction was then extracted from $K_{in}$ to obtain the scalar input stiffness of the RVAPD. Based on the geometric parameters and material properties of the proposed mechanism, the calculated input stiffness was 23,800 N/mm.

The output stiffness was derived using the same flexibility matrix framework, but with the reference coordinate system translated to the center of the working platform. Because the proposed mechanism possesses central symmetry, the two transmission substructures retain an equivalent topological relationship when viewed from the output side. Therefore, the compliance matrices of the flexure hinges were transformed into the output reference coordinate system and combined according to their corresponding series and parallel relationships. The equivalent output compliance matrix of the complete mechanism can consequently be expressed as

(6) $$C_{out}=C_{I}^{O_{out}}+C_{II}^{O_{out}}$$

The different forms of Equations (2) and (6) arise from the distinct load-transfer relationships at the input and output sides: the two substructures act in parallel with respect to the input loading, whereas their output-side compliances contribute through the corresponding deformation compatibility and load-transfer relationship. The equivalent output compliance matrices $C_{I}^{O_{out}}$ and $C_{II}^{O_{out}}$ are obtained by applying the same series–parallel superposition procedure to the flexure hinges within each substructure. The corresponding expressions are given in Equations (7) and (8).

(7) $$C_{I}^{O_{out}}={\left\{\begin{array}{c} {\left[{\left[C_{2}^{-1}+{\left(C_{3}+C_{4}\right)}^{-1}+{\left(C_{5}+C_{6}\right)}^{-1}\right]}^{-1}+C_{19}+C_{7}\right]}^{-1}\\ +C_{20}^{-1}+C_{22}^{-1}+C_{21}^{-1} \end{array}\right\}}^{-1}$$

(8) $$C_{II}^{O_{out}}={\left\{\begin{array}{c} {\left[{\left[C_{25}^{-1}+{\left(C_{23}+C_{24}\right)}^{-1}+{\left(C_{28}+C_{27}\right)}^{-1}\right]}^{-1}+C_{29}+C_{41}\right]}^{-1}\\ +C_{42}^{-1}+C_{43}^{-1}+C_{44}^{-1} \end{array}\right\}}^{-1}$$

The global output stiffness matrix is subsequently obtained by taking the inverse of the equivalent output compliance matrix:

(9) $$K_{out}={\left(C_{out}\right)}^{-1}$$

The stiffness component along the normal direction of the polishing interface was extracted from $K_{out}$ to characterize the resistance of the polishing end against external processing disturbances. The resulting theoretical output stiffness was 8856 N/mm.

### 3.2. Dynamic Modeling Analysis

The natural frequency of the RVAPD is a key dynamic parameter for determining its non-resonant operating range and evaluating the dynamic characteristics of the proposed mechanism. To establish an analytically tractable dynamic model, the compliant transmission mechanism was simplified to an equivalent single-degree-of-freedom mass–spring system. In this model, the distributed masses and rotational inertias of the moving components are converted into an equivalent mass referred to the generalized displacement coordinate, while the overall structural stiffness is represented by an equivalent stiffness. The Lagrange method was employed to derive the dynamic equation of the system.

The input displacement of the PZT-driven mechanism was selected as the generalized coordinate and is denoted by $q=y_{in}$, where $y_{in}$ represents the displacement at the driving input. The corresponding output displacement of the polishing platform is denoted by $y_{out}$. According to the kinematic transmission relationship of the proposed compliant mechanism, the displacement of each moving component can be expressed as a function of the generalized coordinate. Thus, the translational velocity and angular velocity of the *i*-th moving component can be written as

(10) $$\dot{y_{i}}=\frac{\partial y_{i}}{\partial q}\dot{q}$$

(11) $$\omega_{i}=\frac{\partial \theta_{i}}{\partial q}\dot{q}$$

where $y_{i}$ and $\theta_{i}$ denote the translational and rotational displacements of the *i*-th moving component, respectively, and $\dot{q}$ is the generalized velocity.

Based on the Lagrange formulation, the governing equation of the system can be expressed as

(12) $$\frac{d}{dt}\left(\frac{\partial E_{k}}{\partial \dot{q}}\right)-\frac{\partial E_{k}}{\partial q}+\frac{\partial E_{p}}{\partial q}=F_{i}$$

where $E_{k}$ is the total kinetic energy of the system, $E_{p}$ is the potential energy, and $F_{i}$ represents the generalized external force.

For the *i*-th moving component, its kinetic energy consists of translational and rotational contributions and can be expressed as

(13) $$E_{k}=\frac{1}{2}M_{i}v_{i}^{2}+\frac{1}{2}J_{i}\omega_{i}^{2}$$

where $M_{i}$ is the mass of the *i*-th moving component, $J_{i}$ is its mass moment of inertia, $v_{i}$ is its translational velocity, and $\omega_{i}$ is its angular velocity. By substituting the kinematic relationships of the moving components into Equation (13) and summing the kinetic energies of all moving components, the total kinetic energy of the RVAPD can be obtained. The specific mass and geometric parameters used in the calculation are listed in Table 2.

For the proposed mechanism, the total kinetic energy can be expressed as

(14) $$E_{k}=2\times \left(\begin{array}{c} \frac{1}{2}M_{1}\dot{y_{in}^{2}}+\frac{1}{2}\left(M_{2}+M_{3}+M_{4}\right)\dot{y_{2}^{2}}\\ +\frac{1}{2}M_{5}\dot{y_{4}^{2}}+\frac{1}{2}\left(\frac{M_{5}L_{9}^{2}}{12}\right){\left(\frac{\dot{y_{in}}}{L_{9}}\right)}^{2} \end{array}\right)+\frac{1}{2}M_{6}\dot{y_{out}^{2}}$$

By expressing the velocities of the individual moving components in terms of the generalized input displacement $q=y_{in}$, Equation (14) can be reduced to the standard equivalent-mass form

(15) $$E_{k}=\frac{1}{2}m_{eq}\dot{y_{in}^{2}}$$

where $m_{eq}$ is the equivalent mass referred to the input displacement coordinate $y_{in}$. Based on the mass and geometric parameters listed in Table 2 and the corresponding kinematic relationships of the mechanism, the equivalent mass was calculated as 0.35653 kg.

The equivalent stiffness of the RVAPD was obtained from the input force–displacement relationship established in Section 3.1.2. Under the small-deformation and linear-elastic assumptions, the equivalent input stiffness is expressed as

(16) $$K_{eq}=\frac{F_{in}}{y_{in}}$$

where $F_{in}$ is the input force and $y_{in}$ is the corresponding input displacement. According to the stiffness analysis presented in Section 3.1.2, the theoretical input stiffness of the proposed RVAPD is 23,800 N/mm. Therefore, this value was adopted as the equivalent stiffness in the first-order dynamic model.

For the equivalent single-degree-of-freedom mass–spring system, the undamped natural frequency is given by

(17) $$f=\frac{1}{2\pi }\sqrt{\frac{K_{eq}}{m_{eq}}}\approx 1300.35\;Hz$$

This analytical result represents the first-order undamped natural frequency of the idealized compliant transmission mechanism. The PZT actuators, fastening bolts, specimen fixture, and external polishing load were not modeled as independent deformable components or dynamic excitation terms in the analytical model. The PZT excitation is represented through the prescribed input displacement, while the fastening and fixture effects are represented by the corresponding mechanical constraints. The external polishing load is not included in the intrinsic natural-frequency calculation because the analytical model is intended to characterize the unloaded dynamic characteristics of the compliant transmission mechanism. The influence of these practical factors on the actual dynamic response is subsequently evaluated through finite element analysis and experimental testing.

## 4. Finite Element Simulation

To verify the static and dynamic characteristics predicted by the analytical model and to further evaluate the structural performance of the proposed RVAPD, three-dimensional finite element simulations were performed using ABAQUS 2022. The RVAPD was modeled using 7075Al-T6 aluminum alloy (Shenzhen Ying Ding Xin Tool&Die Co., Limited, Shenzhen, China)), with a Young’s modulus of 71.7 GPa and a density of 2810 kg/m^3^. The finite element model was discretized using tetrahedral elements with a free-meshing technique. The global mesh size was set to 6 mm, while the curvature control factor was set to 0.1, corresponding to approximately eight elements per circular curvature. The minimum element size was set to 0.6 mm, corresponding to 10% of the global mesh size. The resulting mesh contained approximately 46,897 elements. The mesh density was maintained sufficiently high in the flexure hinge regions to capture the localized deformation and stress concentration associated with compliant motion. The mesh-generation parameters were selected to balance numerical accuracy and computational efficiency.

For the modal analysis, the structural constraints corresponding to the actual mounting conditions of the RVAPD were imposed, and the undamped natural frequencies and corresponding mode shapes were extracted. For the static stiffness analysis, a force of 1000 N was applied at the corresponding input or output loading locations, and the resulting displacement was used to calculate the input and output stiffness according to the force-to-displacement relationship. The PZT actuators and fastening bolts were not explicitly modeled as deformable components; instead, their mechanical effects were represented through the corresponding loading and boundary conditions [30]. This modeling strategy was adopted to focus the finite element analysis on the compliant mechanism and to maintain consistency with the analytical model, in which the flexure-based transmission structure is the primary load-bearing and deformation component. The finite element model was subsequently used to obtain the first-order natural frequency, input and output stiffness, maximum von Mises stress, and output displacement for comparison with the theoretical predictions.

### 4.1. Modal Analysis

**Figure 5:**
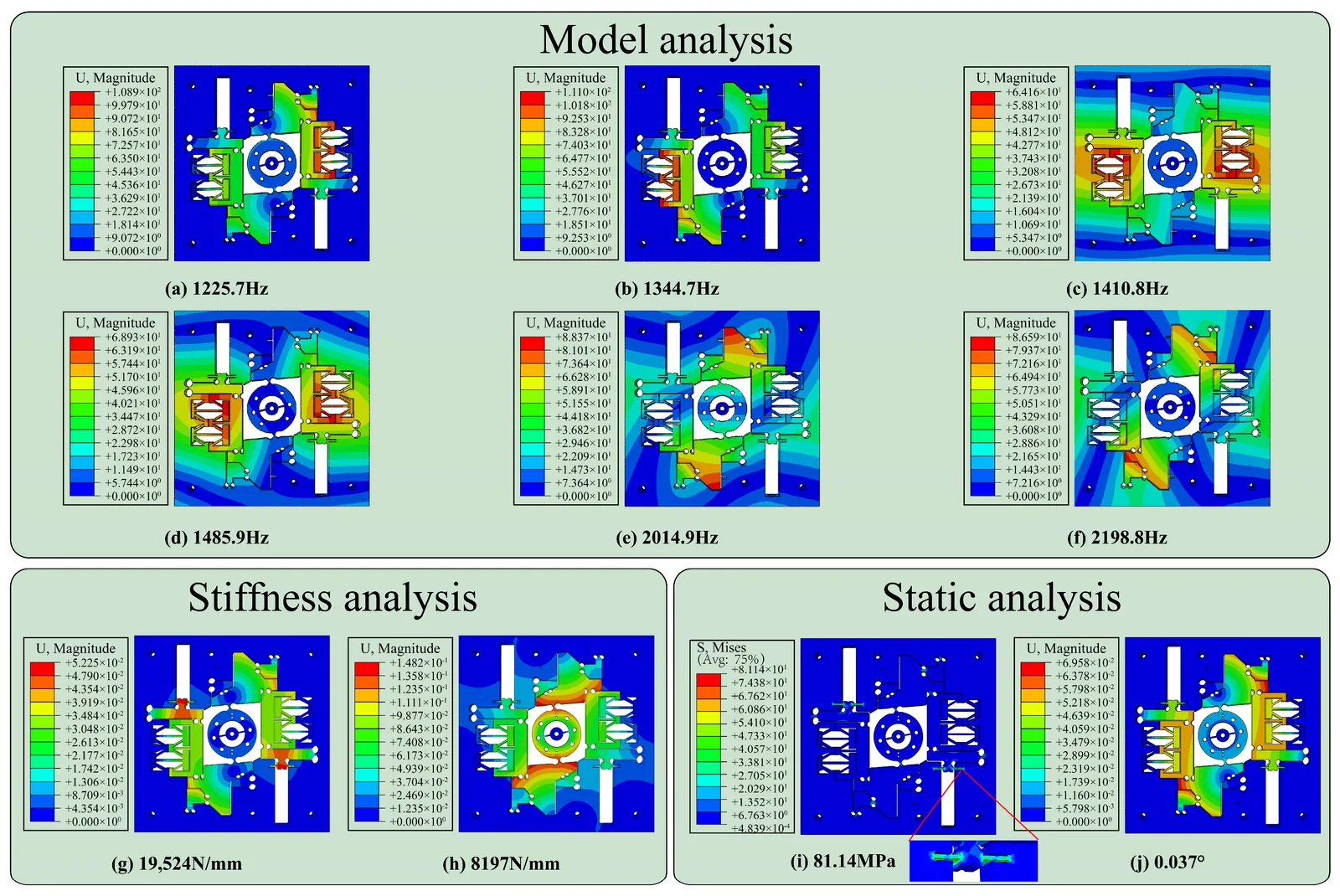
Finite element simulation of RVAPD. (**a**) First-order frequency. (**b**) Second-order frequency. (**c**) Third-order frequency. (**d**) Fourth-order frequency. (**e**) Fifth-order frequency. (**f**) Sixth-order frequency. (**g**) Input stiffness. (**h**) Output stiffness. (**i**) Maximum stress. (**j**) Maximum displacement.

Modal analysis is the core method for delineating the non-resonant safe operating frequency band of the RVAPD and verifying the multi-degree-of-freedom decoupling characteristics of the system. ABAQUS software was used to extract the three-dimensional finite element eigenvalue characteristics of the system; the first six mode shapes are shown in Figure 5a–f. The simulation results indicate that the first six undamped natural frequencies of the system are 1225.7 Hz, 1344.7 Hz, 1410.8 Hz, 1485.9 Hz, 2014.9 Hz, and 2198.8 Hz, respectively; among them, the analytical prediction of the natural frequency has a relative error of only 5.74% compared with the theoretical derivation results in the previous text, indicating good agreement between the analytical model and the finite element result. From the evolution of the modal pattern, it can be seen that the deformation direction of the first-order dominant mode of the system is highly consistent with the expected cutting output trajectory. More importantly, the first-order natural frequency of 1225.7 Hz is substantially higher than the excitation frequencies investigated in this study (100–300 Hz), providing a sufficient frequency separation between the operating range and the first structural resonance. At the same time, there is a significant frequency difference of 119 Hz between the main working mode (first order) and the higher-order parasitic modes (second order and above), achieving excellent band decoupling, ensuring the kinematic purity and trajectory fidelity of the RVAPD under non-resonant alternating loads.

### 4.2. Static Analysis

#### 4.2.1. Finite Element Modal Stiffness

As shown in Figure 5g, when forces of 1000 N were applied in opposite directions on both sides of the RVAPD, the input end moved by 0.0512 mm. Based on the simulation test results from the computer software, the input stiffness of the device was calculated as 19,524 N/mm. This value is lower than the stiffness of the PZT itself, which fully meets the design indicators from the previous stage. Moreover, this simulation result is very close to the previous theoretical calculation results, with a deviation of only 17.97%.

Following the same testing procedure, when a force of 1000 N is applied to both sides of the central platform, a displacement of 0.122 mm will be generated at the output end. From this, the output stiffness of the device can be calculated as 8197 N/mm. The specific details are shown in Figure 5h. This value is not significantly different from the previous theoretical calculation results, with an error of only 7.44%. The specific data comparison of theoretical formula calculation and software simulation testing is summarized in Table 3.

The relatively larger difference in input stiffness (17.97%) compared with the output stiffness difference (7.44%) can be attributed to the differences between the idealized analytical model and the detailed finite element model. In the analytical model, the flexure hinges are represented by equivalent compliance matrices based primarily on their ideal geometric and material parameters, whereas the finite element model directly considers the three-dimensional geometry of the complete compliant structure. In addition, the analytical model assumes ideal series–parallel connections and idealized boundary conditions, while the finite element model includes the actual geometric constraints and load-transfer paths of the assembled structure. These modeling simplifications can lead to differences in the predicted deformation, particularly at the input side where multiple compliant elements and transmission stages contribute to the overall deformation. Therefore, the 17.97% difference should be regarded as a consequence of model idealization rather than as a negligible numerical deviation.

#### 4.2.2. Maximum Stress Analysis

In the actual application of the RVAPD, in order to ensure that the processing operation is reliable and safe, the maximum force that the equipment can withstand must always be kept within the safe range allowed by the material. Otherwise, structural damage may occur. We used ABAQUS software to conduct a simulation analysis of the force situation, and identified the location with the highest stress concentration on the vibration platform. Considering that the yield strength of the 7075Al-T6 material used for the device is 524 MPa, after setting a safety factor of 1.8, the allowable stress of this material under normal operation can be calculated using Formula (12).

(18) $$\left[\sigma \right]=\frac{\sigma_{s}}{s}=291.11\;MPa$$

By referring to Figure 5i, it can be observed that when a force of 1000 N was applied simultaneously to both input ends of RVAPD, the maximum stress $\sigma_{max}$ endured by the device was 81.14 MPa. This value is significantly lower than the material’s safe load-bearing limit. Moreover, the area with the most concentrated force exhibited a relatively thinner LSFH region, indicating that the deformation that occurs during the operation of the entire workbench can naturally recover. Therefore, it is fully capable of performing long-term high-frequency operation.

#### 4.2.3. Maximum Displacement Analysis

The operation principle of the RVAPD can be roughly regarded as a system with a spring and damping (dissipation) effect. The larger the displacement of the piezoelectric actuator, the greater the reaction force. Due to the influence of this load resistance, the actual output stroke of the piezoelectric ceramic will be reduced, and the extent of this reduction is mainly determined by the input stiffness $K_{in}$ of the equipment itself. By using Formula (13) from reference [31], the maximum achievable moving distance L of the piezoelectric ceramic in the face of different resistances can be calculated. In this formula, $L_{0}$ represents the limit stroke without any load, $K_{T}$ represents the stiffness of the piezoelectric ceramic itself, and $K_{V}$ represents the stiffness brought by the external load (in this paper, it is $K_{in}$). After specific calculation, under the current input stiffness conditions, the piezoelectric ceramic can move a maximum of 12.41 µm. If this moving distance is simultaneously applied to both input ends, the maximum rotation angle generated by the central component is 0.037°. The specific deformation situation can be referred to in Figure 5j.

(19) $$L=L_{0}\times \frac{K_{T}}{K_{T}+K_{V}}$$

### 4.3. Harmonic Response Analysis

**Figure 6:**
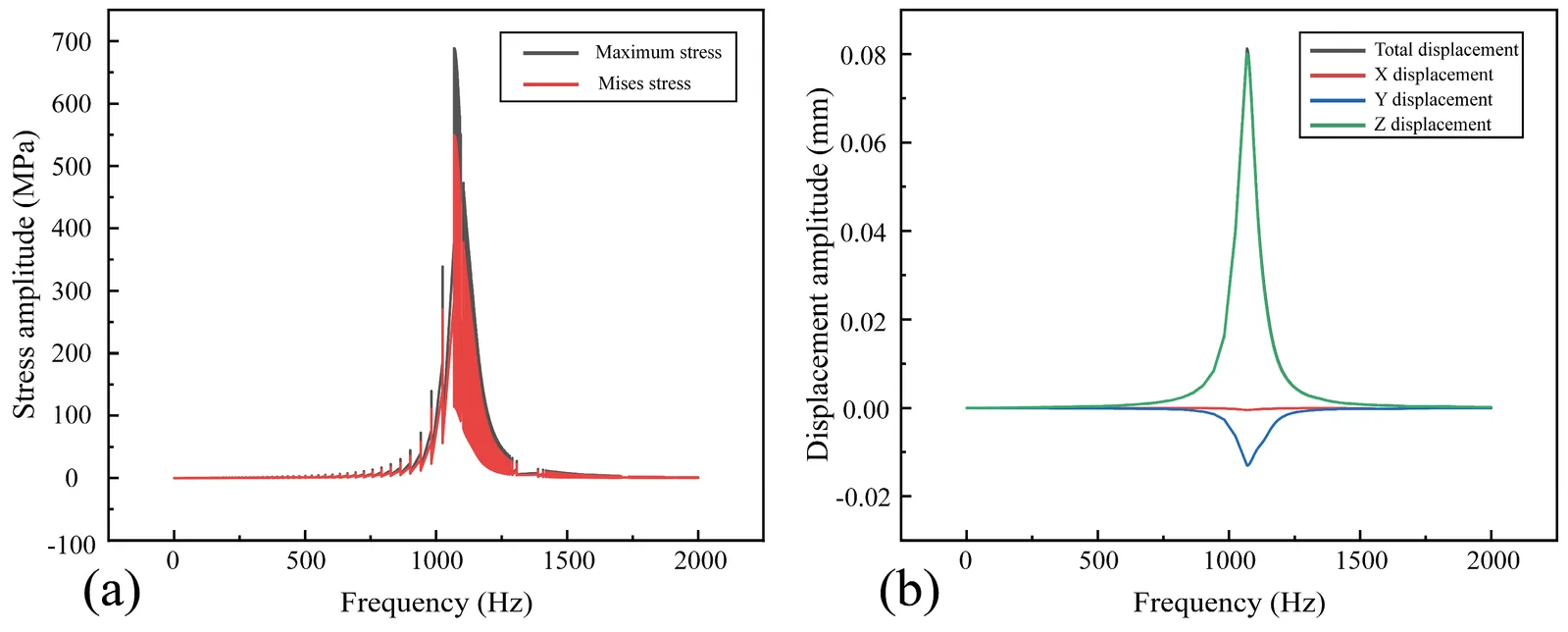
Harmonic response analysis results. (**a**) Stress response. (**b**) Displacement response.

To further evaluate the dynamic response characteristics of the RVAPD under periodic excitation, a harmonic response analysis was conducted over a frequency range of 0–2000 Hz. Two harmonic forces with equal magnitudes and opposite directions were applied to the two input ends, with an amplitude of 1000 N for each force. The frequency-dependent stress and displacement responses of the structure were then obtained, as shown in Figure 6.

As shown in Figure 6a, the stress response remains relatively low over most of the investigated frequency range but increases sharply when the excitation frequency approaches approximately 1.0–1.1 kHz, where a pronounced response peak occurs. A similar frequency-dependent characteristic is observed in the displacement response shown in Figure 6b, with the maximum response appearing in the same frequency region. This consistency between the stress and displacement responses indicates that the pronounced dynamic response is associated with the intrinsic dynamic characteristics of the compliant structure.

In contrast, the excitation frequencies used in the polishing experiments, namely 100, 200, and 300 Hz, are substantially lower than the frequency range in which the pronounced dynamic response occurs. Therefore, the RVAPD operates away from the main dynamic response peak under the investigated polishing conditions. This result supports the non-resonant operating characteristics of the proposed device and indicates that the selected polishing frequencies can generate vibration-assisted motion without intentionally operating near the dominant resonance region.

Furthermore, the harmonic response analysis provides an additional dynamic perspective beyond the modal analysis. While the modal analysis identifies the natural frequencies of the structure, the harmonic response analysis directly describes the stress and displacement responses under periodic loading. The results therefore provide further evidence for selecting the investigated polishing excitation frequencies within the non-resonant operating range.

### 4.4. Mesh Convergence Study

To assess the sensitivity of the finite element results to the mesh density, a study on mesh convergence was conducted using three different global mesh sizes of 8, 6, and 4 mm. All the mesh models maintained the same element type, mesh partitioning strategy, material properties, boundary conditions, and loading conditions. The corresponding minimum element sizes were 0.8, 0.6, and 0.4 mm respectively. The number of elements in the three mesh models was 35, 325, 46,897, and 96,715 respectively.

The first-order natural frequency, input stiffness, output stiffness, and maximum von Mises stress were selected as the main indicators for evaluating mesh convergence. The summarized numerical results obtained under the three mesh densities are shown in Table 4.

As the mesh was gradually refined, the first-order natural frequency decreased from 1384.3 Hz for the 8 mm mesh to 1225.7 Hz for the 6 mm mesh and 1194.3 Hz for the 4 mm mesh. The relative change between the 6 mm and 4 mm meshes was 2.56%, indicating that the predicted natural frequency became progressively stabilized with mesh refinement. Similarly, the input stiffness decreased from 23,255 N/mm to 19,524 N/mm and 18,181 N/mm, corresponding to a relative change of 6.88% between the 6 mm and 4 mm meshes. The output stiffness changed from 8433 N/mm to 8197 N/mm and 7812 N/mm, with a relative change of 4.70% between the 6 mm and 4 mm meshes. Considering the progressive stabilization of the main response parameters and the computational cost associated with further mesh refinement, a 6 mm mesh was selected for the finite element analysis to achieve a practical balance between numerical stability and computational efficiency.

## 5. Performance Testing

### 5.1. RVAPD Physical Prototype

**Figure 7:**
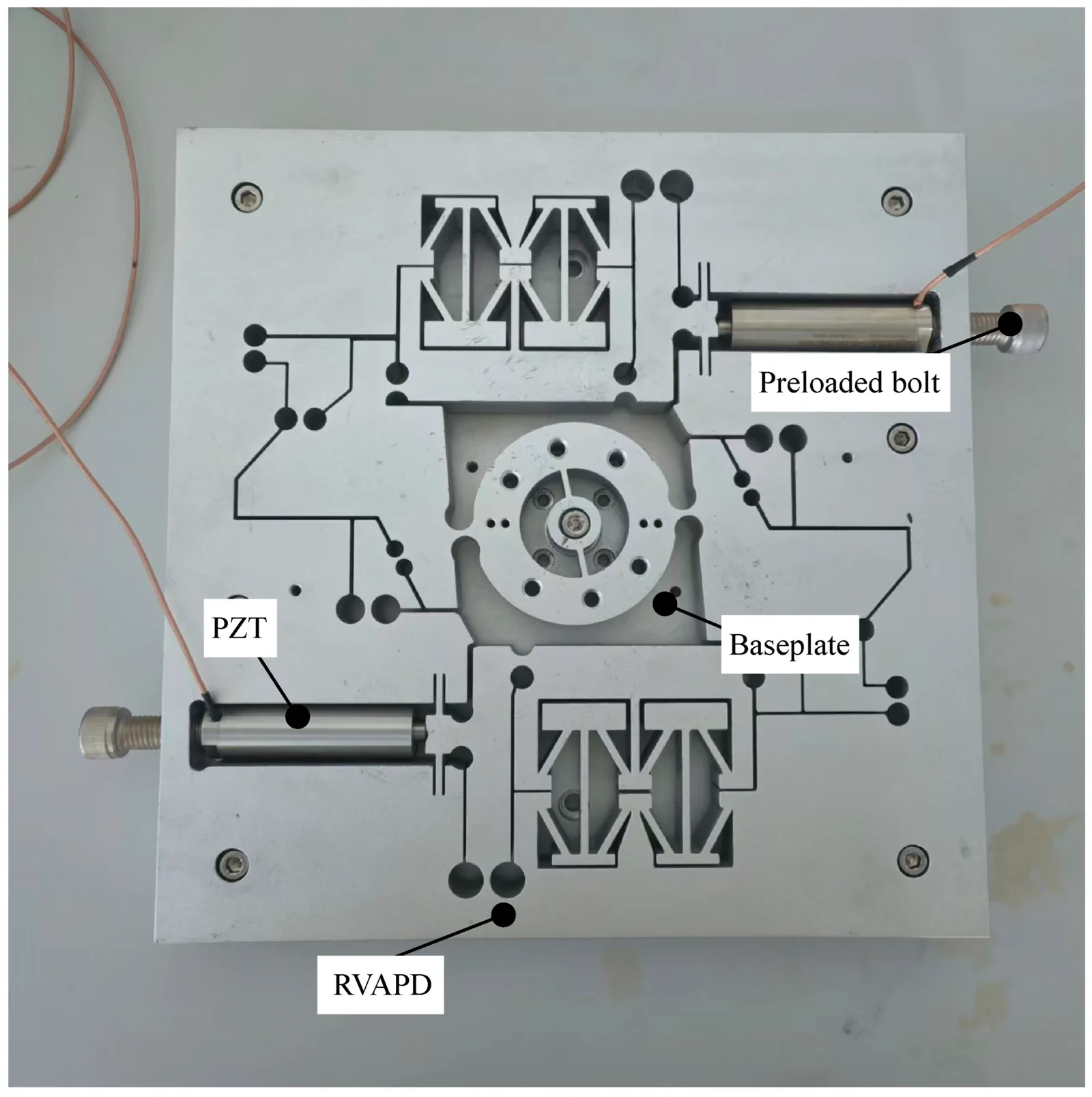
RVAPD physical prototype.

As shown in Figure 7, the physical prototype of the RVAPD was manufactured through wire cutting technology. The device body is mainly made of 7075 Al-T6 aluminum alloy, with a Young’s modulus of 71,700 MPa and a density of 2810 kg/m^3^. During assembly, we placed the piezoelectric ceramic (specific model: PSt150V/10/60VS20 (Harbin Xintian Tomorrow Technology Co., Ltd., (Harbin, China)) into a dedicated groove and fixed it using matching bolts. When a maximum driving voltage of 150 Vpp is applied to it, this piezoelectric ceramic can extend up to 60 μm. In addition, the overall length, width, and thickness of this vibration-assisted platform are 280 mm, 280 mm, and 21 mm respectively.

### 5.2. Work Frequency and Work Space Testing

**Figure 8:**
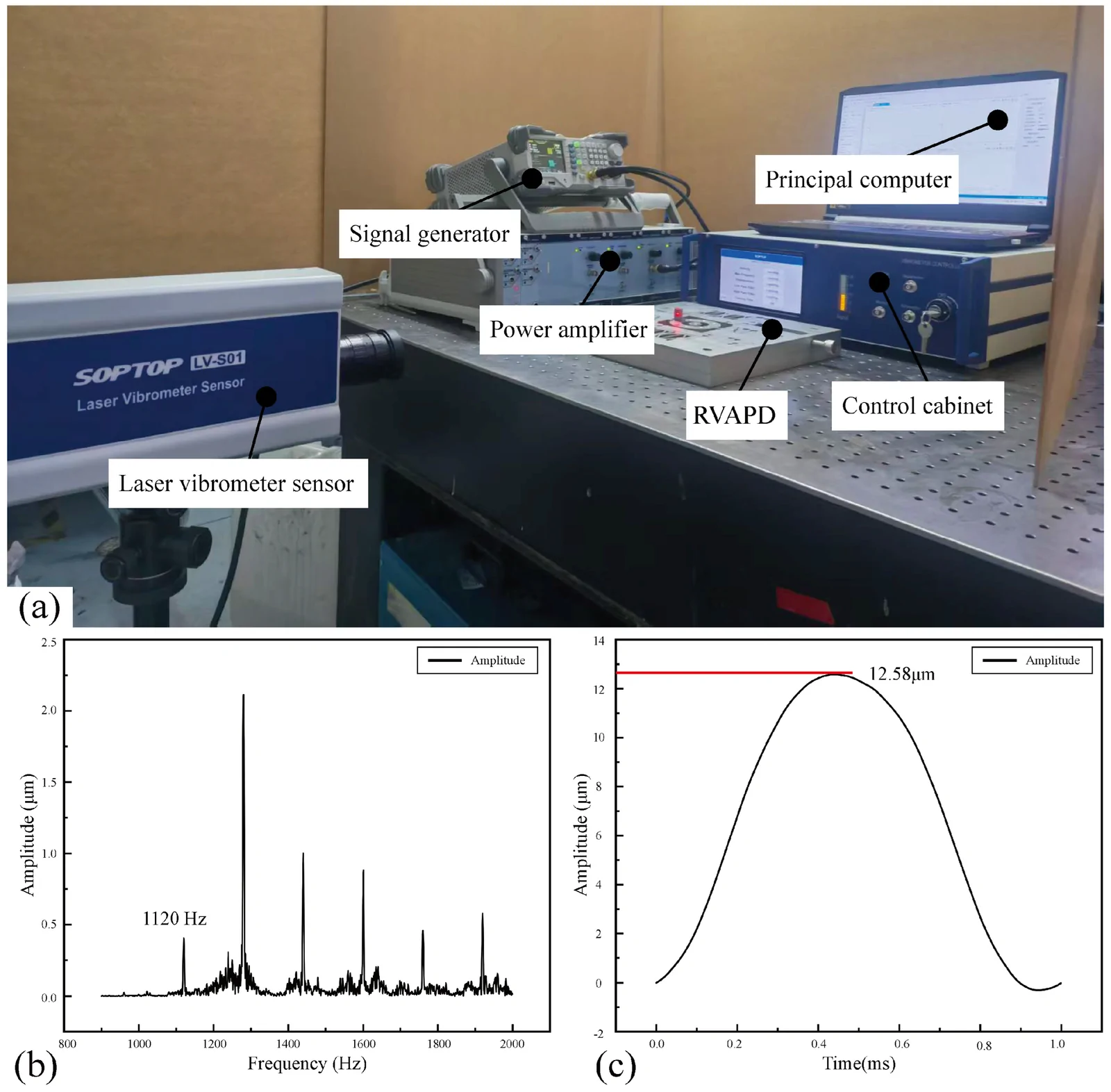
Platform setup and test results. (**a**) Test setup diagram. (**b**) Natural frequency. (**c**) Maximum displacement.

In order to clarify the frequency range and operating range applicable to the RVAPD, we specifically measured its natural frequency and the limit travel, and recorded the offline operation data under different driving voltage conditions. The setup of the relevant testing equipment can be referred to in Figure 8a, and to avoid external interference, the entire experiment was conducted on an air-float vibration isolation platform. During the experimental testing phase, a signal generator was used (Suzhou Puyuan Precision Electronics, model DG1062Z, (Suzhou, China)) to emit two identical signals. These signals were enhanced by a power amplifier (Harbin Xintianming Technology, model E00.C4, (Harbin, China)), and then transmitted to the PZT on both sides, thereby causing the middle platform to rotate. Due to the extremely small actual rotation distance, we used the linear movement along the tangent direction of the platform edge as the final output result. Finally, the laser vibration meter (Zhejiang Sunnyinnovation Optical Intelligence, model LV-S01, (Yuyao, Zhejiang, China)) was responsible for capturing these tiny movement data, and then the control cabinet transmitted them to the computer for unified storage.

In order to determine the actual operating frequency range of the RVAPD, we used a pulse hammer to strike the side of the central platform, allowing it to naturally vibrate. Then, the Laser Vibrometer Sensor was used to capture and record the displacement changes. The specific test results are shown in Figure 8b. The measured natural frequency of the device was 1120 Hz, which is slightly lower than the previous simulation result based on the computer software. This difference was mainly attributed to the assembly method and manufacturing errors, as well as the tiny cumulative errors in each part during the manufacturing process.

The PZT displacement measurement results are shown in Figure 8c. A sinusoidal signal with a frequency of 10 Hz and an amplitude of 10 Vpp was generated by the signal generator and subsequently amplified to 150 Vpp by a power amplifier before being applied simultaneously to the two PZT actuators. Under this excitation condition, the maximum measured displacement of the RVAPD was 12.58 μm, compared with the analytical prediction of 12.41 μm. The measured value was approximately 0.17 μm (1.37%) higher than the predicted value. This small discrepancy may be attributed to differences between the idealized analytical model and the actual experimental system, including assembly tolerances, clamping and preload conditions, manufacturing deviations, and the actual mechanical boundary conditions of the prototype. In addition, the analytical model does not explicitly account for the compliance and contact characteristics of the assembled components. The close agreement between the measured and predicted displacements indicates that the analytical model can reasonably capture the displacement response of the RVAPD under the tested excitation condition.

### 5.3. Output Stiffness Test

#### 5.3.1. Construction of the Testing Platform

**Figure 9:**
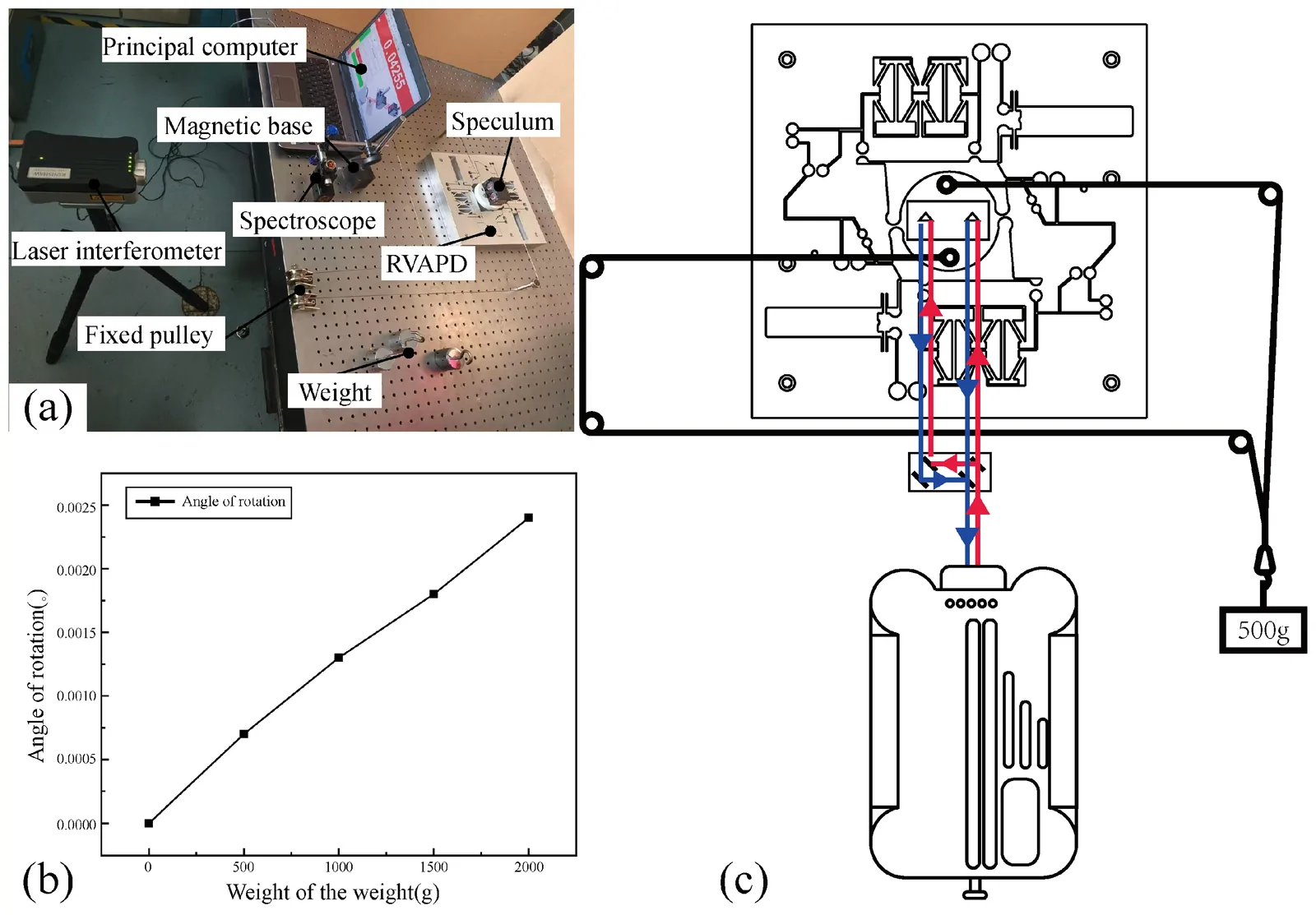
Platform setup and test results. (**a**) Test setup diagram. (**b**) Test principle and optical path. (**c**) Angle test results.

In order to test the actual movement performance of this vibration-assisted equipment and its advantages in resisting external resistance, an output stiffness testing system was developed. The specific details can be referred to in Figure 9a. During the experiment, we applied a continuous and gradually increasing tension to the vibration drive component of the equipment, with a maximum load of 500 g. The tensile force was transmitted through the nylon rope and through the fixed pulley, acting on both sides of the middle workbench, thereby causing the entire platform to rotate. The measurement principle of the output stiffness and the optical path are illustrated in Figure 9c. The XL-80 (Renishaw, PLC, Wotton-under-Edge, UK) laser interferometer emitted a laser beam. This beam, after passing through the splitting component and the mirror cavity of the interferometer, will return. Because the rotation of the platform will cause the reflecting mirror to move, resulting in a change in the distance traveled by the light (the optical path difference), the interferometer precisely records the rotation angle of the central platform by capturing this change in light.

#### 5.3.2. Output the Test Results of Stiffness

The specific test data are recorded in Figure 9b. We set the initial angle to zero (0°). As the weights were successively increased, the measured platform rotation angles reached 0.0007°, 0.0013°, 0.0018°, and 0.0024° respectively. Since the laser interferometer directly measures the angular displacement of the platform, the measured rotation angles were converted into equivalent linear displacements to calculate the output stiffness. The equivalent linear displacement can be expressed as

(20) $$\delta_{out}=r\cdot \theta$$

In the formula, r represents the effective distance from the rotation center to the measurement position, and θ represents the measured rotation angle. According to the experimental setup, r is set to 16.26 mm. Combining the weights attached and these rotation data, we calculated that the actual output stiffness of the equipment was 7200 N/mm. This measured value is lower than the one simulated by the computer. The reason might be that the internal parts of the machine are fixed together by bolts, and how tightly the bolts are screwed will directly affect the overall deformation resistance of the machine body. At the same time, the external pulling force is pulled by the nylon rope to the top of the bolts, thereby driving the platform in the middle and below to rotate. Due to the fact that the bolts used to transmit the pulling force are difficult to make absolutely perpendicular to the bottom air-floating platform, the actual rotation distance of the platform is slightly smaller than the distance pulled by the rope. As a result, the calculated stiffness is also lower than the simulated value. However, the experimental results also prove that although the actual measured stiffness is slightly lower than the theoretical expectation, this vibration-assisted equipment still has a very high rigidity performance. It can ensure that the machine runs smoothly under vibration conditions and meets the strength requirements of high-frequency vibration polishing operations.

### 5.4. Static Performance Testing

**Figure 10:**
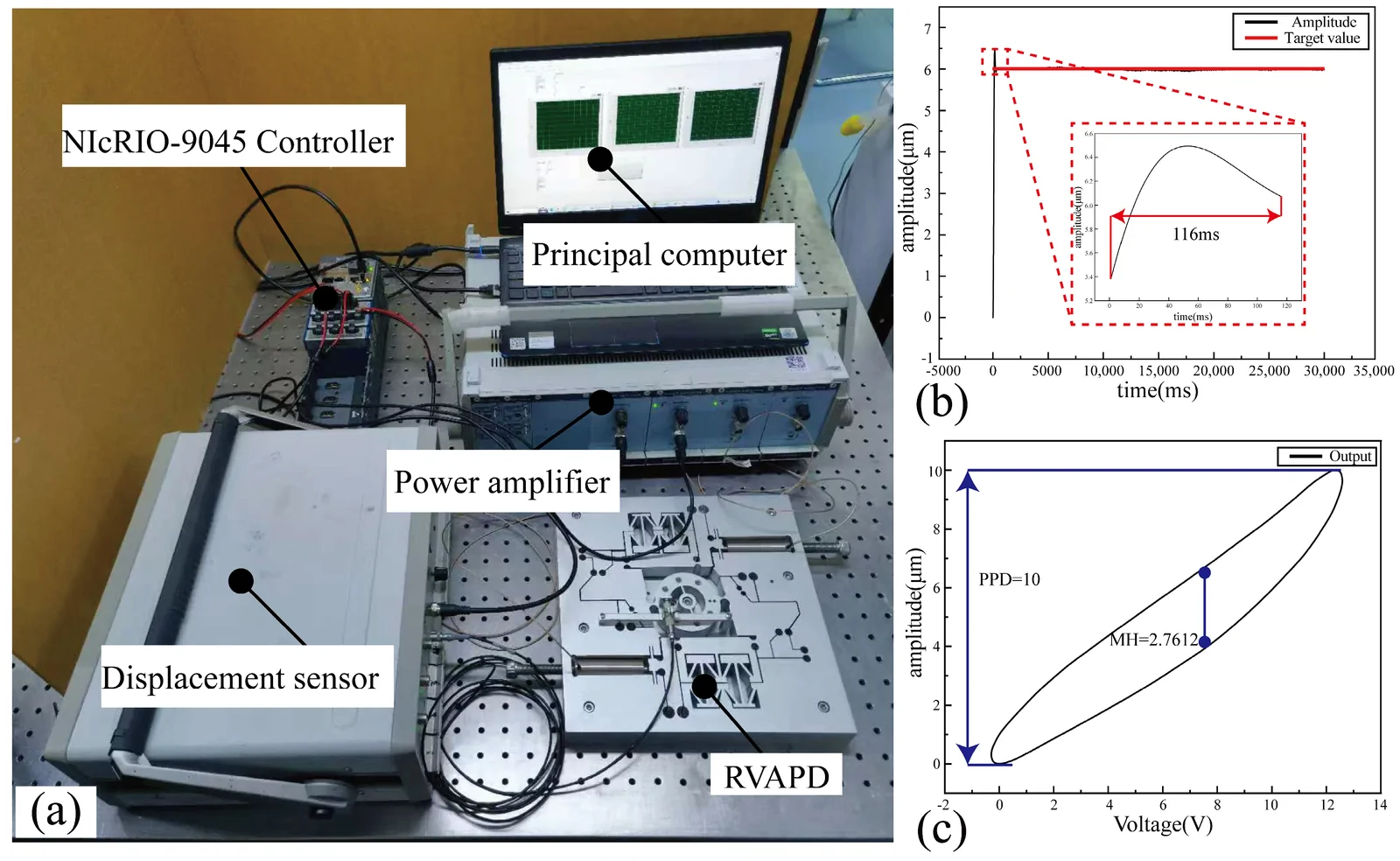
Setup and test results of the static performance testing platform. (**a**) Test setup diagram. (**b**) Step Response test. (**c**) Hysteresis test.

To evaluate the static performance of the RVAPD, we input low-frequency signals into the PZT for driving. In this test, we used the Micro Sense sensor to record the movement of the central workbench, thereby detecting the Step Response and hysteresis phenomenon of the RVAPD. The hardware layout of the entire static performance test is shown in Figure 10a.

To test the positioning accuracy of the RVAPD, we set the target’s movement distance to 6 µm and tested its response speed. The specific process is shown in Figure 10b. During the entire monitoring period of 30,000 ms, after receiving the instruction, the device initially had a 114 ms response delay. Then, at the 143 ms mark, it first reached the target position. During this period, the actual action climb time was only 29 ms. Although there may be a slight position deviation and minor overshoot during the final stabilization of the action, this device can still quickly and accurately execute the control instructions issued by the outside world and can fully meet the actual requirements of the vibration-assisted polishing process.

The hysteresis phenomenon is a characteristic inherent in the PZT, which to some extent reduces the performance and positioning accuracy of the RVAPD [32]. Therefore, conducting specialized hysteresis tests is highly necessary. However, through ingenious and flexible mechanical structure design, this adverse effect can be effectively mitigated. As can be seen from the test data in Figure 10c, when we input a driving command with a frequency of 0.5 Hz and an amplitude of 5 µm, the actual measured total travel of the device was 10 µm. During the test, the displacement error was calculated as the absolute difference between the measured displacement and the commanded displacement at each measurement point, and the maximum positioning error was determined as the maximum value of these errors. The resulting maximum positioning error was 2.7612 μm. This test result fully demonstrates that the currently designed device can fully meet the actual operational requirements of the vibration-assisted polishing process in terms of motion accuracy.

## 6. Vibration-Assisted Polishing Experiment

### 6.1. Construction of the Experimental Platform

**Figure 11:**
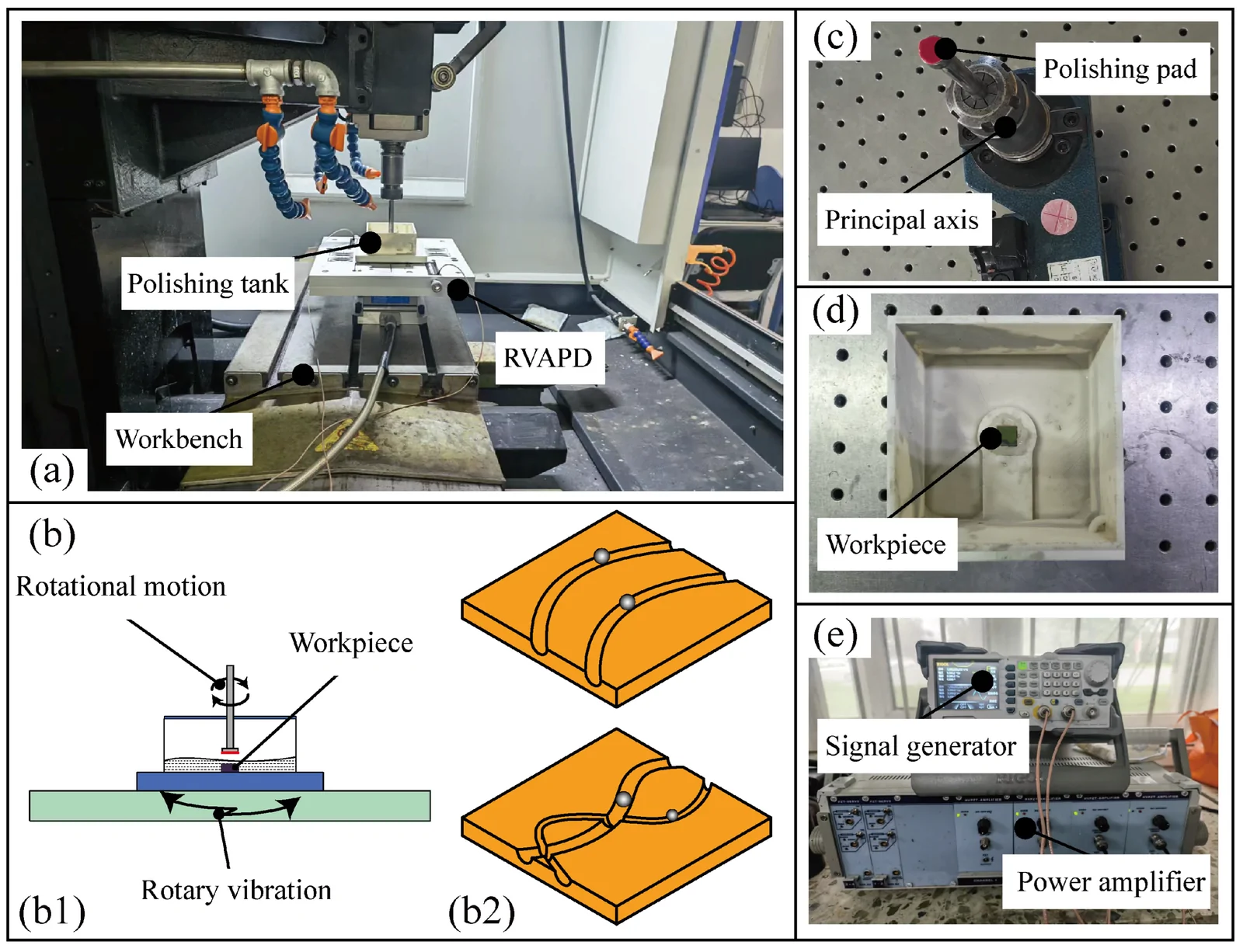
Setup of vibration-assisted polishing experiment and polishing principle. (**a**) Schematic diagram of the experimental platform. (**b**) Schematic diagram of the experiment. (**b1**) Experimental schematic diagram. (**b2**) Comparative diagram of polishing trajectory. (**c**) Details of the polishing head. (**d**) Details of the processing area. (**e**) Signal processing equipment.

The specific vibration-assisted polishing experiment was carried out using a common CNC machine (KEMT-MT52) available on the market. The overall hardware setup is shown in Figure 11a. During the experiment, we directly tightened the RVAPD using bolts onto the operating table of the machine. As for the processing object, it was a 10 mm × 10 mm × 10 mm spherical cast iron specimen. We stuck it with hot melt glue under a 3D-printed base as the main polishing surface. Then, as shown in Figure 11d, the base and the specimen were immersed in a polishing slurry containing 1 μm diamond abrasives (Shenzhen Xin Hui Technology Co., Ltd. (Shenzhen, China)). The polishing pad used for grinding was made of felt and directly glued to the rotating spindle of the machine with glue. The specific construction details can be referred to in Figure 11c. The dynamic process of the entire vibration processing can be intuitively understood through Figure 11(b1). As shown in Figure 11e, the vibration instructions required during operation were specifically transmitted to the RVAPD by the power amplifier. After adding vibration, the originally single scraping routes of the fine abrasive particles in the polishing liquid would interweave and overlap with each other. The specific effect is shown in Figure 11(b2). This change not only made the movement trajectory of the abrasive particles more complex but also significantly accelerated the speed of removing excess material and improved the overall efficiency.

### 6.2. Surface Quality

**Figure 12:**
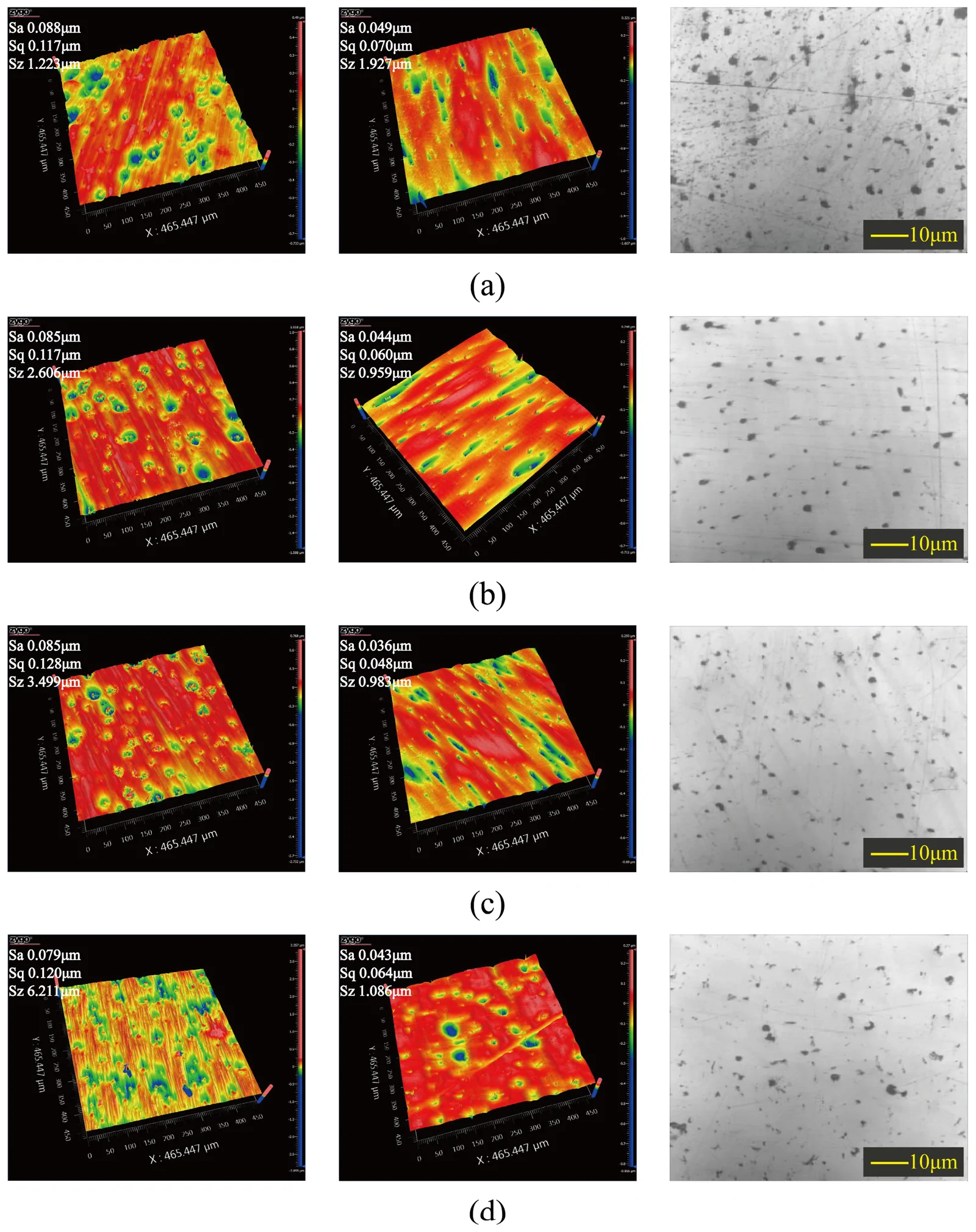
Measurement of surface roughness of ductile iron. (**a**) Without vibration. (**b**) With 100 Hz vibration. (**c**) With 200 Hz vibration. (**d**) With 300 Hz vibration.

To evaluate the effect of vibration assistance on the polishing of nodular cast iron, comparative experiments between vibration-assisted and non-vibration-assisted polishing were conducted. Based on preliminary experiments, the rotational speed, scanning speed, and polishing time were set to 300 rpm, 100 mm/min, and 40 min, respectively. To investigate the effect of excitation frequency under a consistent vibration amplitude, the input voltage of the PZT actuators was individually adjusted at each excitation frequency. Different input voltages were applied at 100, 200, and 300 Hz to maintain approximately the same vibration amplitude at the polishing platform. This approach minimized the influence of amplitude variation and enabled a direct comparison of the polishing performance at different excitation frequencies. Three repeated polishing experiments were conducted under each experimental condition, and the reported surface roughness values are presented as the mean ± standard deviation (SD). Under the non-vibration condition, as shown in Figure 12a, although the areal surface roughness (Sa) decreased from 0.088 μm to 0.0497 ± 0.0021 μm, the maximum height (Sz), which characterizes extreme local surface defects, increased from 1.223 μm to 1.570 ± 0.352 μm, accompanied by the appearance of dense dark pits in the 2D images. With the introduction of vibration assistance, both the density and depth of the observed surface defects were reduced. Among the tested excitation frequencies of 100, 200, and 300 Hz as shown in Figure 12b–d, the best surface-quality results were obtained at 200 Hz, at which the final surface Sa reached 0.0363 ± 0.0015 μm and the Sz value decreased to 0.985 ± 0.010 μm. When the excitation frequency was increased to 300 Hz, the surface-quality indicators showed a slight deterioration, with Sa increasing to 0.0457 ± 0.0025 μm and minor cluster-like surface pits reappearing in the 2D images. Among the tested excitation frequencies of 100, 200, and 300 Hz, 200 Hz demonstrated the most favorable polishing performance, with the lowest mean Sa and Sz values. The subsequent deterioration at 300 Hz confirms that the polishing response is sensitive to the excitation frequency. Thus, 200 Hz represents the most favorable excitation condition within the investigated frequency range.

The improvement in surface quality can be attributed to the changes in abrasive–workpiece interactions induced by vibration assistance. Previous studies have shown that vibration-assisted polishing can modify the motion and contact state of abrasive particles, thereby affecting material removal and polishing-pad wear [33]. In the present experiments, the introduction of vibration changed the surface morphology from dense scratches and pronounced deep pits to a more uniform surface with substantially fewer and shallower local defects. This behavior indicates that vibration altered the material-removal process rather than simply increasing the overall polishing intensity. Specifically, the superimposed vibration continuously changes the relative motion between the abrasive particles and the workpiece, resulting in more varied abrasive trajectories and a dynamically varying contact condition. Such changes can redistribute the local contact pressure and material-removal intensity, thereby reducing excessive removal concentrated at specific locations. Meanwhile, the dynamic relative motion can facilitate the displacement of detached debris from the polishing zone, reducing the likelihood of repeated abrasive interactions with previously generated defects. These effects provide a reasonable explanation for the observed reduction in the density and depth of surface pits, as reflected by the decreases in Sa and Sz. The pronounced reduction in Sz is particularly significant because Sz is strongly affected by isolated deep defects; therefore, the decrease in Sz from 1.927 μm under non-vibration polishing to 0.983 μm at 200 Hz indicates that vibration assistance effectively reduced the severity of extreme local surface defects. At 300 Hz, however, Sa increased to 0.043 μm and localized cluster-like pits reappeared, further demonstrating that the material-removal behavior is dependent on the excitation frequency. Overall, the experimental observations support the interpretation that vibration assistance modifies abrasive trajectories and contact conditions and thereby promotes a more uniform material-removal process. Although the present white-light interferometry and optical microscopy primarily characterize the resulting surface morphology, the observed reduction in surface defects is consistent with the proposed changes in abrasive motion and contact behavior. The present results therefore provide experimental evidence that vibration assistance modifies the abrasive–workpiece interaction state and improves the uniformity of material removal, while further microstructural characterization is needed only to resolve the specific subsurface and interfacial damage mechanisms.

## 7. Conclusions

This paper presents a two-dimensional non-resonant flexible vibration-assisted polishing device (RVAPD) based on a series–parallel structure and the Scott Russell mechanism. The spatial motion and mechanical transmission are decoupled through an irregular flexible hinge network. The theoretical, simulation, and experimental results show that the simulated input and output stiffnesses are 19,524 N/mm and 8197 N/mm, respectively, while the experimentally measured output stiffness reaches 7200 N/mm. The measured natural frequency is 1120 Hz, the maximum output displacement is 12.58 μm, and the static response delay is 114 ms. Among the tested excitation frequencies of 100, 200, and 300 Hz, 200 Hz demonstrated the most favorable polishing performance, with Sa decreasing to 0.036 μm and Sz decreasing to 0.983 μm, compared with Sa = 0.049 μm and Sz = 1.927 μm under the corresponding non-vibration condition. At 300 Hz, the surface-quality performance showed a slight deterioration, with Sa increasing to 0.043 μm and localized cluster-like pits reappearing. The subsequent deterioration at 300 Hz indicates that the polishing response is sensitive to the excitation frequency. Thus, 200 Hz represents the most favorable excitation condition within the investigated frequency range. The observed surface morphology indicates that vibration assistance can modify the abrasive–workpiece interaction state and promote a more uniform material-removal process. The reductions in surface-defect density and depth, together with the decrease in Sz, are consistent with the effects of vibration on abrasive trajectories, dynamic contact conditions, and local material-removal behavior. These results demonstrate the potential of vibration assistance to reduce localized surface damage and improve the surface quality of ductile cast iron, providing experimental support for the application of the proposed RVAPD in vibration-assisted polishing of multiphase heterogeneous materials.

## Figures and Tables

**Table 1:** Quantitative comparison between Ref. [24] and the present RVAPD.

Parameter	Ref. [24]	Present Work	Quantitative Difference
Main transmission mechanism	Hoecken linkage	Scott Russell mechanism	Topology redesigned
Target mater	SiC ceramic	Ductile cast iron	Extended to multiphase heterogeneous material
Theoretical input stiffness	51,052 N/mm	23,800 N/mm	−53.4%
Theoretical output stiffness	6145.8 N/mm	8856 N/mm	+44.1%
Experimental output stiffness	4454 N/mm	7200 N/mm	+61.6%
Experimental natural frequency	1220 Hz	1120 Hz	−8.2%
Main design objective	Rotary vibration polishing of hard/brittle SiC	High-stiffness non-resonant vibration polishing of multiphase ductile cast iron	Application and structural objective extended

**Table 2:** Parameters for equivalent mass and lever arm length used.

M_1_	M_2_	M_3_	M_4_	M_5_	M_6_	L_9_
0.0642 kg	0.0760 kg	0.0086 kg	0.0160 kg	0.1379 kg	0.0560 kg	70.14 mm

**Table 3:** Results of theoretical calculations and finite element simulations.

	Input Stiffness	Output Stiffness	Inherent Frequency
Simulated analysis	19,524 N/mm	8197 N/mm	1225.7 Hz
Model analysis	23,800 N/mm	8856 N/mm	1300.35 Hz
Error	17.97%	7.44%	5.74%

**Table 4:** Mesh convergence results for the RVAPD.

Mesh	Elements	Frequency	Input Stiffness	Output Stiffness
8 mm	35,325	1384.3 Hz	23,255 N/mm	8433 N/mm
6 mm	46,897	1225.7 Hz	19,524 N/mm	8197 N/mm
4 mm	96,715	1194.3 Hz	18,181 N/mm	7812 N/mm

## Data Availability

The data presented in this study are available on request from the corresponding author.
